# Speakers Use Indirect Language to Pursue Their Own Goals While Managing Social Relationships

**DOI:** 10.1162/OPMI.a.388

**Published:** 2026-09-05

**Authors:** Nicole Gotzner, Gregory Scontras

**Affiliations:** Cognitive Science Institute, Osnabrück University, Osnabrück, Germany; Department of Language Science, University of California, Irvine, United States

**Keywords:** indirect language, pragmatic inference, politeness, negation asymmetry, negative strengthening

## Abstract

We test three theories of the use and interpretation of indirect language by manipulating speaker interaction styles, focusing on an interpretation asymmetry that has puzzled philosophers and grammarians since Greek antiquity. We observe that negated evaluatively-positive (*not happy*) and evaluatively-negative (*not sad*) adjectives are interpreted asymmetrically for a speaker who regularly uses loopholes. Our results provide evidence that speakers use the loophole opened up by the indirectness of negation to plausibly deny an evaluatively-negative meaning. In contrast, the predicted interaction effects of competing politeness and Pollyanna-based theories are not borne out by our experiments. We interpret these results as evidence that loopholes are the key mechanism driving the asymmetric interpretation of negated statements. We also find evidence that hearers take into account that the speaker protects their own self-image rather than just mitigating a potential face-threat posed towards the hearer. Overall, our results suggest that speakers use indirect language to pursue their own goals while managing their social relationships.

## INTRODUCTION

Communication is often driven by efficiency considerations. By relying on the hearer to enrich what is said, speakers can leave communicated content implicit (Grice, 1989). But speakers also use seemingly inefficient language for social reasons. For example, when your friend says *You don’t look happy*, what they likely want to convey is that you look sad. Yet saying *You look sad* directly risks coming across as blunt or hurting you. The indirect *not happy* softens the message, allowing your friend to express a concern without causing offense. The current work investigates the benefits of using such indirect language for the speaker, the hearer, and their social relationship. We do so by testing a well-described phenomenon that has puzzled logicians, philosophers, and grammarians since Greek antiquity—the asymmetric interpretation of negated adjectives. When evaluatively-positive adjectives are used with a negation, as in *You don’t look happy*, they are more likely to mean the opposite (that you look sad), while negated negative adjectives like in *You don’t look sad* are interpreted as middling statements (e.g., Gotzner & Mazzarella, 2021; Mazzarella & Gotzner, 2021; Ruytenbeek et al., 2017). These meanings do not strictly follow from the logical effect of negation on meaning (Horn, 1989); hearers are enriching the literal meanings by reasoning pragmatically about what the speaker intended to communicate.

There is consensus in the literature that speakers use negated statements for pragmatic purposes, but the underlying reasoning principles have been subject to debate. The current work tests three different theories that have been proposed in the literature. Speakers may use negated statements (i) to leave themselves a *loophole* (Gotzner & Scontras, 2024; Krifka, 2002; Lee & Pinker, 2010; Seright, 1966), (ii) for the sake of *politeness* (Brown & Levinson, 1987; Horn, 1991), or (iii) to simply avoid straightforwardly-negative expressions (e.g., Mazzarella & Gotzner, 2021; Terkourafi, Weissman, & Roy, 2020; building on the *Pollyanna* Principle from Boucher & Osgood, 1969).

Humans have a broad range of communicative strategies in order to not fully commit to the content that is communicated by the words they are using. Saying *You don’t look happy* allows the speaker to weasel out of the negative content the hearer may infer. For example, if you openly challenged your friend by saying *I am not sad*, your friend could deny they ever wanted to suggest this (plausible deniability, e.g., Lee & Pinker, 2010; Pinker et al., 2008). Such a *loophole* strategy is especially useful when the communicated meaning contains negative evaluations; the strategy is less appropriate when the communicated content contains positive evaluations (Gotzner & Scontras, 2024). Hence, according to the *loophole theory*, negated statements are interpreted asymmetrically because the hearer recognizes the speaker’s incentive to remain vague about what they wanted to communicate exactly in situations of potential conflict. In this sense, speakers are using indirect language strategically in order to maintain plausible deniability in a broad range of social phenomena (Lee & Pinker, 2010).

*Politeness theory* explains the asymmetric interpretation via the need of interlocutors to maintain face, that is, a public image (Brown & Levinson, 1987; Goffman, 1955; Horn, 1989). According to the classic politeness-based explanation, saying *You look sad* directly would threaten the hearer’s face, and therefore the speaker chooses an indirect statement, using negation to mitigate the face threat. Positive statements (*You look happy*), in turn, do not threaten the hearer’s face in most situations. Thus, politeness theory assumes that hearers recognize the face-threatening potential of different expressions the speaker could have chosen (see Gotzner & Mazzarella, 2021, for experimental evidence). So, essentially speakers are taken to not want to use expressions that hurt the hearer’s feelings, which is a pattern hearers recognize. In this case, the hearer strengthens the meaning of the negated expression to mean *sad*, thus this inference has been called “negative strengthening” (Horn, 1989). In more recent work by Yoon et al. (2020) within the Rational Speech Act framework, politeness is modeled via competing social goals. On this view, indirect language is used to fulfill the speaker’s desire to maintain a public image and to appear as prosocial. Thus, indirect language is taken to have both a prosocial and a self-presentational utility. Our experiment tests the classic politeness-based explanation of negative strengthening. We will come back to the Yoon et al. modeling framework in our discussion.

Finally, there is a general human tendency to focus on the bright side of life, known as the *Pollyanna* principle (Boucher & Osgood, 1969). This principle is reflected in positive expressions being used more frequently than negative ones across languages (Dodds et al., 2015). Accordingly, speakers may avoid saying negative evaluations directly because it makes them feel better (Mazzarella & Gotzner, 2021; Terkourafi et al., 2020). On this view, speakers do not use negated statements to safeguard the hearer’s face but rather to remain positive. Therefore, hearers should recognize that the speaker was not in a position to utter a more positive statement. While the classic Pollyanna principle applies to a preference for simple positive expressions, an application of this Principle to indirect language takes speakers to prefer semantically-positive expressions (potentially for self-concerned reasons; see Mazzarella & Gotzner, 2021; Terkourafi et al., 2020).

While the Pollyanna view focuses on the speaker’s desire to remain on the bright side of life, the politeness view focuses on the desire to not make the hearer sad. The loophole theory synthesizes these two functions and payoffs of indirect language via one mechanism: by remaining vague, speakers can potentially retract a communicated meaning if openly challenged, thereby protecting their own reputation and their social relationship. Loopholes are particularly useful to navigate situations of conflict and manage the social relationship between speakers and hearers (Gotzner & Scontras, 2024; Pinker et al., 2008). Previous work has provided evidence for the role of plausible deniability in indirect language through judgments of speaker’s intent (Lee & Pinker, 2010) and ratings that link strengthened interpretations to adjective subjectivity (Gotzner & Scontras, 2024) (operationalizing loopholes as the potential for faultless disagreement).

All three of the existing theories are consistent with the results of previous experimental studies that have corroborated the asymmetric interpretation of negated adjectives (e.g., Gotzner & Mazzarella, 2021; Mazzarella & Gotzner, 2021; Ruytenbeek et al., 2017). The main empirical gap is that previous work has not directly tested what role a speaker’s interaction style plays in hearer’s reasoning strategies and to what extent the hearer’s interpretation shifts as a function of the speaker’s goals.

To tease apart the predictions of the three theories, the current study introduces an experimental manipulation of speaker interaction styles, based on different accounts. Doing so allows us to assess the role of loopholes, politeness, and Pollyanna-based considerations in the interpretation of negated statements. Our experiments were set up in a way that each theory predicts a polarity effect in a critical condition but not the respective control condition. Table 1 summarizes the principles assumed in the different theories, on which side of the communication coin they are taken to apply, and the predictions each theory makes regarding our experiments. In the loophole experiment, the critical speaker communicates via loopholes while the control speaker is committed to the implicated meaning. If the hearer recognizes that loopholes are useful in situations of conflict, then we should find a polarity asymmetry for the loophole speaker but not the committed speaker. In the politeness experiment, the critical polite speaker safeguards the hearer’s face while the control self-concerned speaker is concerned about their own face. If the polarity asymmetry arises via politeness considerations regarding the hearer’s face, then we should find such an asymmetry for the polite but not the self-concerned speaker. Finally, in the Pollyanna experiment the critical speaker is always positive while the control speaker is always negative. If the polarity asymmetry arises due to the speaker’s desire to remain positive, we should find a polarity asymmetry for the positive but not the negative speaker.

**Table 1.** Summary of different theories of negative strengthening, the principle they assume, on which side of the communication coin this principle operates, alongside our experimental manipulation.

	**Loophole**	**Politeness**	**Pollyanna**
Principle	remain vague	avoid face threat	stay positive
Side	speaker (and hearer)	hearer	speaker
Manipulation	loophole (vs. committed)	polite (vs. self-concerned)	positive (vs. negative)

## METHODS

### Participants

For each experiment, we recruited 60 English-speaking participants based in the U.S. on Prolific; participants were only allowed to take one of the experiments. We pre-screened participants for English as their native language, an age range between 18 and 67, and the nationality and current country of residence being the United States. Per our pre-registration, participants who answered 4 or more of the filler trials incorrectly were excluded from further analyses (loopholes experiment: 4 participants, politeness experiment: 8, and Pollyanna experiment: 6). Ethics approval was granted by the Ethics Commission of the DGfS (Deutsche Gesellschaft für Sprachwissenschaft; vote nr. 2019-17-210202). Participants gave informed consent before the start of the experiment and all procedures performed were in accordance with the Declaration of Helsinki.

### Items and Task

We developed three experiments with a 2 polarity (positive vs. negative) x 2 speaker interaction style (control vs. critical) design. For each experiment, we adapted the experimental materials from Mazzarella and Gotzner (2021), who pretested the stimuli for several linguistic criteria (see Appendix A in Mazzarella & Gotzner, 2021, for more information). For each item, the type of speaker who made the statements was described (see Figure 1). These interaction styles were exemplified in the experiment introduction (see Figure 3). All stimuli and experiment introductions for individual experiments are available at OSF and the supplement presents the materials for one experimental condition. Participants saw the same polarity–speaker interaction style combinations over the course of an experiment. For example, a participant in the loopholes experiment might see the committed speaker produce negated positive adjectives and the loophole speaker produce negated negative adjectives, while another participant would see the committed speaker produce negated negative adjectives and the loophole speaker produce negated positive adjectives.

**Figure 1.**
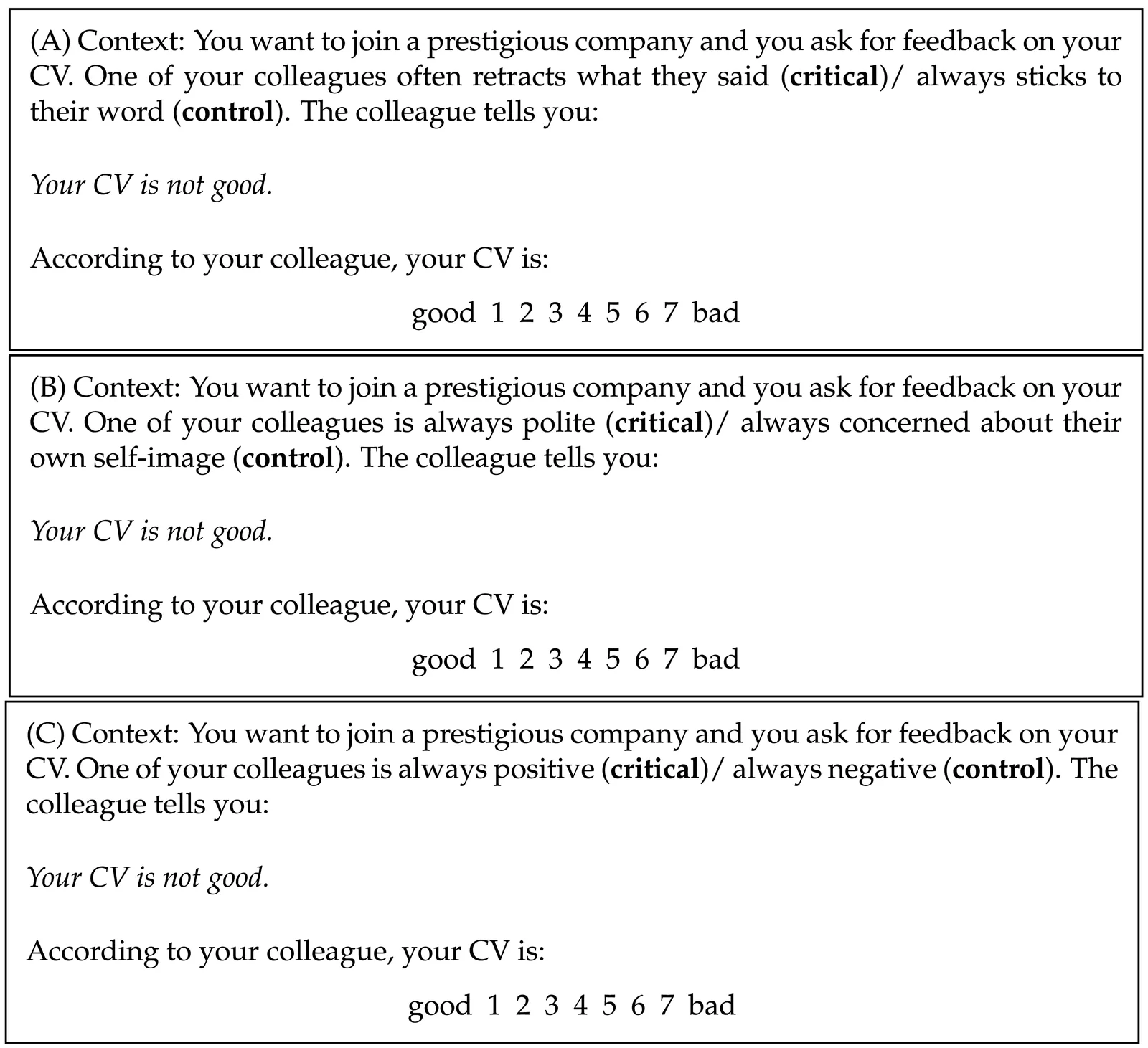
Example trial for a positive adjective in the loophole experiment (A), politeness experiment (B), and Pollyanna experiment (C).

The experiments were set up in a way that the three theories predict a polarity effect in the critical but not the control conditions. This nested interaction effect was pre-registered for the individual experiments: loophole, politeness, pollyanna. In the loophole experiment, we compared a critical loophole speaker to a control committed speaker. Participants were presented with an example where the loophole speaker denied the implicated content of a statement they had made, whereas the committed speaker confirmed the implicated content. Thus, our manipulation tapped into plausible deniability and speaker commitment. In the politeness experiment, we compared a critical polite speaker to a control self-concerned speaker. The polite speaker was presented to be concerned about safeguarding other people’s feelings, whereas the self-concerned speaker protected their own self-image (and was said to not be concerned with other people’s feelings). In this way, the politeness manipulation contrasts the speaker’s desire to save their own vs. the listener’s face. In the Pollyanna experiment, we compared a critical positive speaker to a control negative speaker. Here, the positive speaker was said to always be positive (saying *amazing* in a mediocre situation in the experiment introduction) whereas the negative speaker was said to always be negative (saying *terrible* in a mediocre situation). Figure 1 presents an example experimental trial for each experiment.

After encountering a statement with a negated adjective, the participants’ task was to judge what meaning the speaker intended to convey (e.g., whether, according to the speaker, *the CV is not good* conveys that CV is good vs. bad). We used a seven-point Likert scale with endpoints labeled with the non-negated adjective (e.g., *good*, value 1) used in the original statement and the corresponding negative antonym (*bad*, value 7). Higher ratings indicate a greater degree of negative strengthening, that is an inference towards the antonym (Ruytenbeek et al., 2017). This measure has been used in a number of previous studies that have shown that negated positive adjectives are more likely to be strengthened than the corresponding negated antonyms (Gotzner & Mazzarella, 2021; Mazzarella & Gotzner, 2021). Previous work also compared binary and Likert scale measures (Ruytenbeek et al., 2017), as well as negated forms to simple expressions (Alexandropoulou & Gotzner, 2024). The main pattern was qualitatively similar across different measures, with negated positive adjective being strengthened and negated negative adjectives receiving middling interpretations.

Participants judged 20 antonym pairs embedded in a context with a statement about the type of speaker they were encountering.^1^ In total, participants completed 40 experimental trials, in addition to 10 filler trials that served as attention checks. The items were rotated across conditions and split across two experimental lists. A given participant saw 20 positive adjectives in one speaker condition and the negative version with the other speaker condition. For this reason, the maximal random effects structure justified by our design includes random slopes by item but not the full structure with random slopes by participant. We excluded the data from participants who answered four or more filler trials incorrectly.

## RESULTS

### Pre-registered Null Hypothesis Significance Testing

We carried out three independent experiments that manipulated the interaction style of the speaker who made the critical statements. These interaction styles were exemplified in the experiment introduction, and each experimental trial indicated which style speaker participants interacted with (see Figure 1). Participants in our experiments judged whether the speaker wanted to communicate the opposite meaning, that is the degree of negative strengthening.

Figure 2 shows the ratings across adjective and speaker conditions. For each experiment, the respective theory predicts a nested interaction: the polarity effect should be present in the critical but not the control conditions. To test these predictions, we fit separate cumulative link models with sum-coded contrasts for polarity and speaker type, nested contrasts for the effect of polarity under the speaker condition, and the maximal converging random effects structure justified by our design.^2^ The nested contrasts reflect our hypotheses regarding the presence or absence of a polarity effect for different speaker types (see Schad et al., 2020). These hypotheses and analyses were pre-registered (see the links presented in the methods section). Table 2 summarizes the predicted effects and the experimental findings. The data and analysis script are available at OSF.

**Figure 2.**
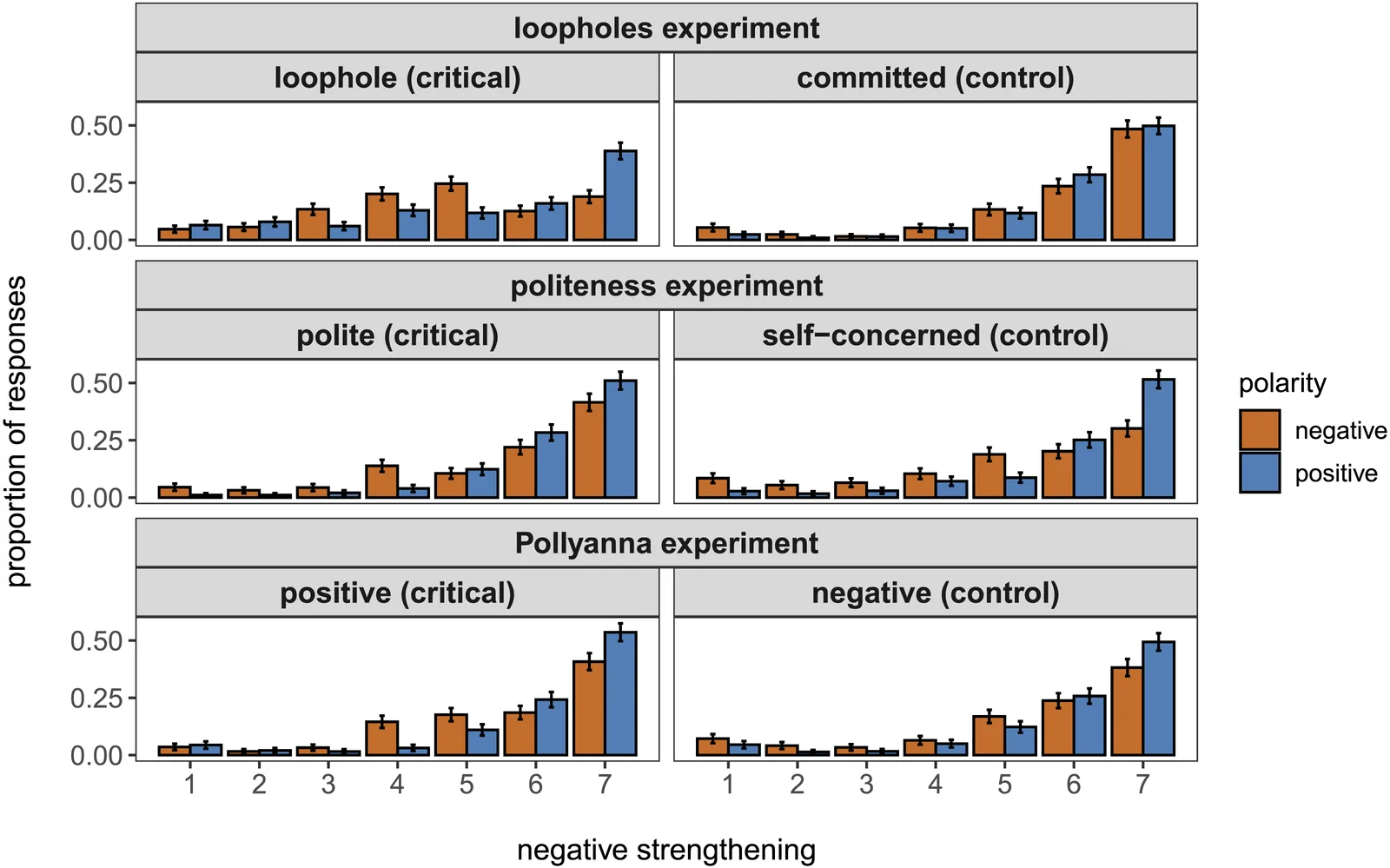
Negative strengthening ratings for positive and negative adjectives in the loopholes, politeness, and Pollyanna experiments. The critical comparison is between the distribution of ratings for positive and negative conditions. Each theory predicts a polarity asymmetry in the critical but not the control conditions.

**Table 2.** Summary of predicted effects for each experiment, together with findings; 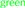 findings are in line with our predictions while 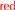 findings are not.

	**Loophole** (critical vs. control)
Predicted effect	loophole 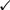 vs. committed 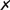
Finding	loophole 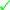 vs. committed 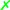
	**Politeness** (critical vs. control)
Predicted effect	polite 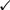 vs. self-concerned 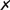
Finding	polite 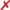 vs. self-concerned 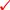
	**Pollyanna** (critical vs. control)
Predicted effect	positive 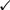 vs. negative 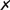
Finding	positive 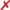 vs. negative 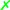

The model for the loophole experiment shows a main effect of the speaker manipulation: the degree of negative strengthening is higher for committed compared to loophole speakers (*β* = −0.86, *z* = −5.74, *p* < 0.01). This effect indicates that participants endorse pragmatic inferences more strongly with the committed speaker type. Crucially, the polarity effect is only significant in the loophole speaker condition but not the committed speaker condition (loophole:polarity *β* = 0.56, *z* = 2.02, *p* < 0.05; committed:polarity *β* = 0.09, *z* = 0.32, *p* = 0.75). This result is in line with the prediction of the loophole theory.

The model for the politeness experiment shows a main effect of the speaker manipulation: the degree of negative strengthening is higher for polite vs. self-concerned speakers (*β* = 0.26, *z* = 2.40, *p* < 0.05). The model shows no polarity effect for the critical polite speaker (*β* = 0.37, *z* = 1.20, *p* = 0.23) but for the self-concerned speaker (*β* = 0.81, *z* = 2.69, *p* < 0.01). This nested contrast is the opposite of what politeness theory predicts.

The model for the Pollyanna experiment shows no significant effect of polarity in the critical positive speaker (*β* = 0.49, *z* = 1.65, *p* = 0.10) nor the control negative speaker (*β* = 0.54, *z* = 1.83, *p* = 0.07). We do not find evidence that Pollyanna-based motivations critically affect the polarity asymmetry in negative strengthening.

### Bayesian Hierarchical Models

In addition to the pre-registered analyses reported above, we also analyzed the results of all three experiments together. This was done via Bayesian hierarchical regression using brms (Bürkner, 2017) in order to quantify the evidence for the hypotheses given the data. The model included sum-coded contrasts for experiment, speaker, and polarity, the nested interaction contrast of polarity in a given speaker type, nested under experiment, as well as the maximal random effects structure justified by the design. We use a cumulative probit linking function since the dependent variable is ordinal. We specify regularizing priors for coefficients with a normal distribution (mean 0, standard deviation 2) (e.g., Bürkner, 2017; Nicenboim et al., 2025). This prior does not introduce a bias regarding the direction of the effect but helps with stabilizing Bayesian estimates and avoiding implausible parameter estimates (a prior predictive check is available on OSF.). The model ran for 10,000 iterations. All Rhat values of the fitted model are 1.00, indicating model convergence. All effective sample sizes are sufficiently high and range between 1,022 and 3,317 (Bürkner, 2017, recommends ESS > 1,000 for stable estimates).

The full set of estimated effects (*β*) and their associated 95% credible intervals (CIs) for each parameter are presented in Table 3. If the 95% credible interval includes zero under the posterior distribution, then we consider zero to be within the range of credible values given the model, the data, and our assumptions (Gelman et al., 1995; Kruschke, 2018). If the interval does not include zero, we interpret this as evidence for an effect given the model and the data. Our model provides evidence for the presence of the following effects: (1) an effect of speaker type within the loophole experiment (critical less negative strengthening than control; *β* = −0.40, 95 % CrI = [−0.52, −0.28]) with a 100% probability of being negative, (2) the predicted polarity asymmetry for the critical speaker within the loophole experiment (*β* = 0.31, 95 % CrI = [0.01, 0.61]) with a 97.95% probability of being positive, and (3) a polarity asymmetry for the control (self-concerned) speaker in the politeness experiment (*β* = 0.45, 95 % CrI = [0.15, 0.75]) with a 99.85% probability of being positive.

**Table 3.** Posterior estimates and 95% credible intervals from the Bayesian model.

	Estimate	95% CrI Lower	95% CrI Upper
experiment1 (loopholes)	−0.18	−0.39	0.03
experiment2 (polite)	0.05	−0.16	0.26
experimentloopholes:speaker1	−0.40	−0.52	−0.28
experimentpolite:speaker1	0.11	−0.03	0.25
experimentpollyanna:speaker1	0.05	−0.08	0.18
experimentloopholes:speakercritical:polarity1	0.31	0.01	0.61
experimentpolite:speakercritical:polarity1	0.24	−0.07	0.55
experimentpollyanna:speakercritical:polarity1	0.25	−0.05	0.55
experimentloopholes:speakercontrol:polarity1	0.07	−0.23	0.37
experimentpolite:speakercontrol:polarity1	0.45	0.15	0.75
experimentpollyanna:speakercontrol:polarity1	0.27	−0.04	0.58

The other estimates included zero within the 95% CrI, and so we cannot reject the null hypothesis for these estimates.

We also tested for the evidence ratios in favor of null hypotheses for our critical interactions between speaker type and polarity (using the hypothesis function in the brms package). We find strong evidence in favor of the null hypothesis for the polarity effect in the control (committed) speaker in the loophole experiment (evidence ratio of 12.85), moderate evidence in favor of the null hypothesis regarding polarity in the critical speaker in the politeness experiment (evidence ratio of 4.10), and moderate evidence in favor of the null hypotheses regarding polarity for both the critical and control speakers in the Pollyanna experiment (evidence ratio of 3.46 and 3.15, respectively). For the loophole critical speaker and the polite control speaker, the hypothesis test indicated that the value (i.e., 0) lies outside of the 95% credible interval, thus suggesting that the null hypothesis should be rejected.

This Bayesian analysis leads us to conclude that the predicted interaction is borne out in the loopholes experiment but not in the Pollyanna and politeness experiments. For the politeness experiment, we have evidence for a credible effect in the control condition and evidence consistent with the null hypothesis in the critical condition. For the Pollyanna experiment, the analysis provides moderate evidence towards accepting the null hypothesis in both speaker conditions. The relevant code for the analysis, as well as prior/posterior predictive checks and model convergence diagnostics can be found on the OSF repository.

## DISCUSSION

The current study tested three theories of the use and interpretation of negated statements by manipulating speaker interaction styles. The results showed that the predicted interaction effect is only borne out for the loophole manipulation but not the politeness and Pollyanna manipulations. This finding indicates that hearers calibrate their interpretation of negated statements via the speaker’s use of loopholes: the avoidance of commitment to the implicated meaning. Speakers have an incentive to use loopholes to avoid negative meanings, as loopholes allow them to plausibly deny this meaning when openly challenged (see also Gotzner & Scontras, 2024; Lee & Pinker, 2010; Pinker et al., 2008; Qian et al., 2024). Our experimental results suggest that loopholes are the key mechanism driving the asymmetric interpretation of negated statements.

We further find evidence that a speaker who is concerned about their own face elicits a larger asymmetry than a speaker who is concerned for the hearer’s face. The polarity effect for the speaker who is concerned about their own self-image indicates that self-presentational aspects play a role in negative strengthening. This can be understood in terms of the speaker’s desire to manage their reputation, as assumed in work on plausible deniability (Lee & Pinker, 2010). Thus, this finding is consistent with the loophole account. A key insight of the classic politeness framework is that when speakers go through the trouble of using a complex expression (e.g., one including negation), this effort is perceived as polite and tactful. To explain the asymmetry in negated statements, the classic explanation due to Brown and Levinson (1987) postulates that hearers recognize the speaker’s desire to not hurt their feelings when negative evaluations are involved. Contrary to this prediction, we find that, for the polite speaker, there is no clear polarity asymmetry, whereas the statements of speakers that are concerned about their own self-image are interpreted asymmetrically. The direction of the effect makes sense as the stakes are higher when the speaker is concerned about their own face. Further, this result does not indicate that the hearer’s face is irrelevant. However, the classic politeness-based explanation of the polarity asymmetry is at best incomplete as it does not capture the larger role of the speaker’s face. Our findings do not rule out that there are contexts where speakers use negation for politeness reasons. After all, we are finding some evidence for a main effect of speaker type where polite speakers are taken to communicate more indirectly.

However, our experiments indicate that considerations of the hearer’s face do not drive the asymmetry in negative strengthening alone. Instead we find that the asymmetry is more likely to occur for speakers that safeguard their own image. As mentioned above, this effect can be readily explained under the loophole theory, which assumes that speakers use indirect speech to weasel out of a potential conflict. This view therefore synthesizes the different functions of indirect speech and argues that it is optimal for the speaker, the hearer, and their relationship to communicate indirectly when negative evaluations are involved. In this sense, our findings are also compatible with the RSA account by Yoon et al. (2020), which construes politeness as the optimal tradeoff of safeguarding the hearer’s face and managing self-presentational concerns of the speaker. We conclude that speakers use negated statements also for a need to appear prosocial and to manage their own reputations, rather than just avoiding the potential upset of the hearer. Hearers seem to recognize this motivation, but the extent to which they can dissociate different speaker intentions (e.g., being genuinely prosocial or managing their own reputation) is a question for future research.

Our findings do not disprove the Pollyanna view altogether but they indicate that Pollyanna-based motivations do not drive the asymmetry in negative strengthening. This view predicts that the polarity asymmetry arises via the speaker’s need to be positive. However, we are finding no robust evidence for a polarity asymmetry—neither for positive nor for negative speakers. We cannot exclude that another manipulation that clearly dissociates the preference for surface-level positive expressions and semantically-positive expressions would yield different results. Still, we conjecture that a more critical aspect is that the Pollyanna principle is an account of explaining language production behavior, that is the speaker’s general preference for using positive expressions, but less so an account of hearer’s considerations. In summary, our results do not argue against politeness or Pollyanna considerations in indirect language use, but rather suggest that the loophole account provides a better explanation for the polarity *asymmetry* than the politeness or Pollyanna accounts: we find that hearers take into account the speaker’s use of loopholes in their interpretation of negated statements. Overall, our work suggests a much broader underpinning of indirect speech: Speakers use loopholes to pursue their own goals while managing their social relationships. This strategy allows them to weasel out of a potential conflict and thereby safeguard their own face, as well as the hearer’s face. In this sense, our loophole theory synthesizes the different assumptions that have been made about the use and interpretation of negated adjectives.

Different disciplines studying language and communication traditionally focus on either the role of language in information exchange (for example work in Pragmatics, following Grice, 1989), truth-conditional aspects of meaning (work in Semantics such as Partee et al., 2012), or the social dimension of language (work in sociolinguistics; Brown, 2015; Eckert, 2012). To fully understand how people use language, these approaches need to be integrated. Recent work has proposed integrated models for the relation between linguistic meaning and the social world (see for example Burnett, 2023). The current study argues for a similar integrated view. Through language, humans not only exchange information implicitly or explicitly but, at the same time, they construct social identities and relationships. Speakers are not only concerned about their interlocutors; they also pursue self-presentational goals. To conclude, indirect language is a powerful tool that fulfills the multi-faceted needs arising in human communication.

## Acknowledgments

We thank Berit Reise, Minou Ahmadian, Mozhdeh Harisini, Simay Münevver, and Özge Aras for help implementing the experiments.

## Funding Information

The current work was supported by the DFG (Grant numbers 44160701 and 523308575).

## Author Contributions

N.G.: Conceptualization, Project administration, Formal analysis, Writing – original draft, Writing – review & editing. G.S.: Conceptualization, Writing – review & editing.

## Data Availability Statement

The experimental data and analysis script are available at the following OSF repository: https://osf.io/xv95p/files/osfstorage.

## Notes

^1^ For some items, face-related concerns for the larger institution (e.g., the company) might play a role.However, this is not the case for all stimuli.^2^ The full model:
rating ∼ speaker/polarity + (1+polarity/speaker|item) + (1+polarity|participant).


## Figures and Tables

**Figure 3.**
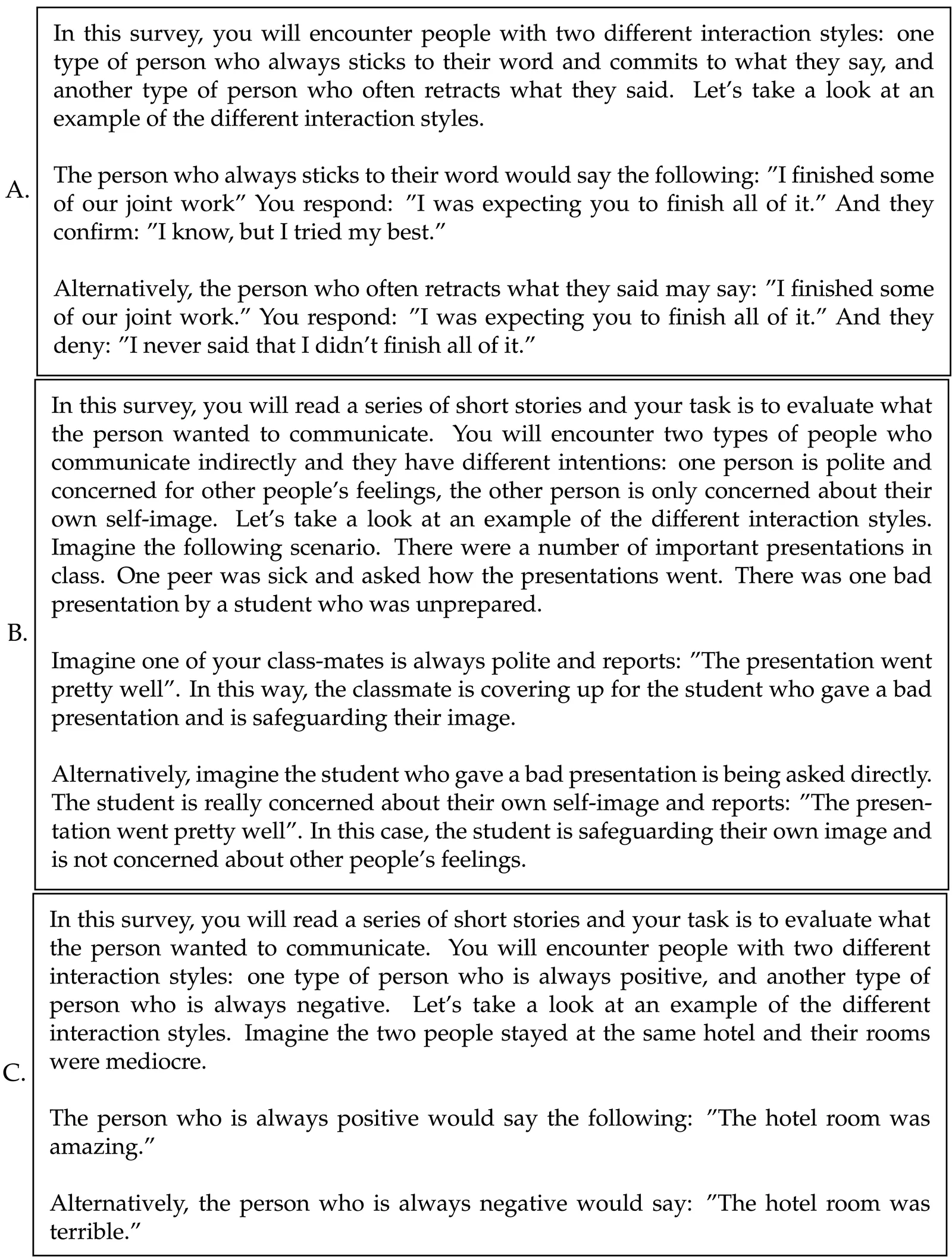
Experiment introductions for the loophole experiment (A), politeness experiment (B) and Pollyanna experiment (C).
